# Hospital librarians as publishers: expanding our role

**DOI:** 10.5195/jmla.2018.541

**Published:** 2018-10-01

**Authors:** Devica R. Samsundar, Carrie Figueredo

**Affiliations:** Director, Library and Research Support, Baptist Health South Florida, Miami, FL; Manager, Library & Research System, Baptist Health South Florida, Miami, FL

## Abstract

Librarians at Baptist Health South Florida (BHSF) launched the first peer-reviewed journal from BHSF, *Nursing & Health Sciences Research Journal (NHSRJ).* This article discusses how it was developed, the role of the librarians, and future goals of the journal.

Baptist Health South Florida (BHSF) is a not-for-profit health care organization that operates numerous hospitals, outpatient centers, and outreach programs in south Florida. The Library and Knowledge Services Department includes five site libraries and provides library services and electronic resources to employees and affiliated physicians system-wide. In March 2018, we launched the first peer-reviewed journal from BHSF, *Nursing & Health Sciences Research Journal (NHSRJ)*. *NHSRJ* provides an open access platform for health care providers to disseminate research findings and improve patient care (Figure 1).

**Figure 1:**
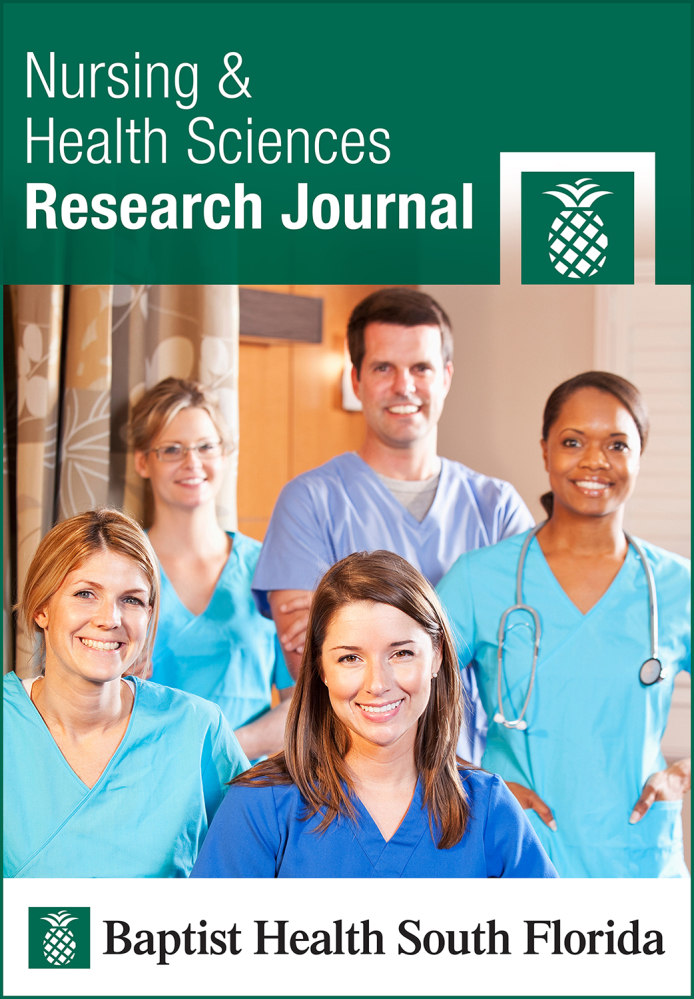
Nursing & Health Sciences Research Journal (NHSRJ)

In 2015, we began discussions with stakeholders at BHSF about the possibility of launching a peer-reviewed journal. The technical capability to provide publishing support services was already in place because the platform for our existing institutional repository, Digital Commons from bepress, includes a publishing module. It includes out-of-the box tools to manage the entire article submission, peer-review, and acceptance process, along with the tools needed to host an online journal. The platform is cloud-based; is highly customizable in look, feel, and organization; and does not require coding skills beyond basic hypertext markup language (HTML).

Since the platform was already budgeted for and licensed by our department and we had recently added staffing to support the project with the hire of a full-time medical librarian, we felt confident that we had the resources necessary to offer this exciting new service line. There is a high level of interest in research dissemination at BHSF, given that several of our hospitals have Magnet status and the high volume of research projects conducted by our health care professionals and staff system-wide. Therefore, we were not surprised by the enthusiastic response that we got from various stakeholders.

The first to partner with the library was the nurse scientist team from the Nursing and Health Sciences Research Department. The library serves as publisher in this partnership. We provide the platform to host and archive the e-journal and all associated support services including copyright advice, training, search engine optimization, journal and site layout, metadata creation, loading of content, and application for an International Standard Serial Number (ISSN). Future plans include obtaining digital object identifies (DOI) assignments to identify journal content and submitting applications for indexing in applicable databases. The nurse scientist team and editorial board is responsible for editorial and submission policies, engagement in marketing communications, and all content, including solicitation of submissions, management of the peer-review process, and copyediting.

While increasingly common in university library settings, providing publishing services remains rare in hospital libraries that are not university affiliated. We enjoyed the opportunity to join our university counterparts and learn about the technical aspects of publishing a journal. We also gained a deeper knowledge of editorial best practices and learned through benchmarking and experience about the importance of clearly defining the scope of services and in drafting service-level agreements for our stakeholders. The result of offering this new service line is that we have raised our visibility and found a new way to demonstrate our value to our organization. The feedback has been overwhelmingly positive, and we look forward to continuing to support publications at BHSF.

